# Supplementary material for: Content-rich biological network constructed by mining PubMed abstracts
Source: BMC Bioinformatics. 2004 Oct 8;5:147. doi: 10.1186/1471-2105-5-147 (PMC528731; doi:10.1186/1471-2105-5-147)
Supplement: Additional File 5 — The original Chilibot query results of the term "long-term potentiation (LTP)" and 22 other terms, limiting the latest references analyzed to the years 1990, 1995, 2000, and 2004. [file 1471-2105-5-147-S5.bz2 › chilibotAdditionalFile5/ltp2004/html/SYNAPSIN I_ACTIN.html]

 


 **SYNAPSIN I** and **ACTIN** 
  
Found 53 abstracts in PubMed,  **30 abstracts were retrieved and analyzed**.  


---

 Search Google  |
 PDF files only 
|  EDU domain only 

---

**Interactive relationship** (e.g. stimulation, inhibition, etc)

- While all the anti  **synapsin I**  mAbs were unable to affect the interactions of  **synapsin I**  both with calcium calmodulin dependent protein kinase II and with  **actin**  monomers and filaments, mAbs 19.8 and 19.51 were found to inhibit the binding of Grb2 SH3 domains to the proline rich C terminal region of  **synapsin I** .  Ref: 9450672 Brain Res Mol Brain Res, 1997
- After training, pp60c src from hippocampus showed enhanced interactions with synaptic proteins such as  **synapsin I** , synaptophysin, and the type 2 N methyl d aspartate receptor, as well as the cytoskeletal protein  **actin** .  Ref: 10884433 Proc Natl Acad Sci U S A, 2000
- SdpII binds dynamin I, synaptojanin,  **synapsin I** , and the neural Wiskott Aldrich syndrome protein, a stimulator of Arp2 3 induced  **actin**  filament nucleation.  Ref: 10704453 J Cell Biol, 2000
- The SH3 mediated interaction of  **synapsin I**  with amphiphysins affected the ability of  **synapsin I**  to interact with  **actin**  and synaptic vesicles, and pools of  **synapsin I**  and amphiphysin I were shown to associate in isolated nerve terminals.  Ref: 10899172 J Biol Chem, 2000
- The results indicate that the interactions of synapsin II with  **actin**  are similar but not identical to those of  **synapsin I**  and suggest that synapsin II may play a major structural role in mature and developing nerve terminals, which is only partially overlapping with the role played by  **synapsin I** .  Ref: 9517476 Eur J Neurosci, 1997
- The fraction of total  **actin**  polymerized by  **synapsin I**  strongly depends on the  **synapsin I**   **actin**  ratio.  Ref: 8305899 J Physiol Paris, 1993
- **Synapsin I**  is a synaptic vesicle specific phosphoprotein which is able to bind and bundle  **actin**  filaments in a phosphorylation dependent fashion.  Ref: 1317863 J Biol Chem, 1992
- **Synapsin I**  is a neuron specific phosphoprotein which binds to small synaptic vesicles and  **actin**  in a phosphorylation dependent fashion.  Ref: 8305899 J Physiol Paris, 1993
- Neurotrophins stimulate phosphorylation of  **synapsin I**  by MAP kinase and regulate  **synapsin I**   **actin**  interactions.  Ref: 8622996 Proc Natl Acad Sci U S A, 1996
- We demonstrated that the head middle region of  **synapsin I**  binds the NH2 terminal domain of beta subunit of calspectin, which also contains an  **actin**  binding site.  Ref: 9070909 Biochem Biophys Res Commun, 1997
- Upon phosphorylation with MAP kinase,  **synapsin I**  showed reduced F  **actin**  bundling activity, while no significant effect on the interaction was observed with the protein phosphorylated with Cdk5 p23.  Ref: 8702879 J Biol Chem, 1996
- Using video enhanced microscopy, we have now obtained experimental evidence in support of this model the presence of dephosphorylated  **synapsin I**  is necessary for synaptic vesicles to bind  **actin** .  Ref: 7876313 J Cell Biol, 1995
- Our findings suggest that  **synapsin I**  exerts a control on the physical characteristics of the cytoskeletal network of the nerve terminal and are consistent with the proposed role of  **synapsin I**  in mediating the interaction of synaptic vesicles with  **actin** .  Ref: 8305899 J Physiol Paris, 1993
- **Synapsin I** , which has been demonstrated to bundle F  **actin**  in vitro, has been postulated to regulate neurotransmitter release by cross linking synaptic vesicles to the  **actin**  cytoskeleton.  Ref: 7931311 J Neurochem, 1994
- The models obtained under various ionic conditions reveal that  **synapsin I**  interacts with  **actin**  in a very complex fashion, sharing some of the properties of classical nucleating proteins but displaying also actions not described previously for other  **actin**  binding proteins.  Ref: 1597463 J Biol Chem, 1992
- Coordinated regulation of  **synapsin I**  interaction with F  **actin**  by calcium calmodulin and phosphorylation inhibition of  **actin**  binding and bundling.  Ref: 7849051 Biochemistry, 1995
- the cross linking between synaptic vesicles and  **actin**  is specific for the membrane of synaptic vesicles and does not reflect either a non specific binding of membranes to the highly surface active  **synapsin I**  molecule or trapping of vesicles within the thick bundles of  **actin**  filaments.  Ref: 7876313 J Cell Biol, 1995
- Thus, the observed decrease in F  **actin**  staining in infected cells might be ascribed to an indirect action of rabies nucleocapsid on the effects of  **actin**  binding proteins such as  **synapsin I** .  Ref: 9367369 J Gen Virol, 1997
- The inhibition of organelle movement in axoplasm by  **actin**  binding proteins such as DNase I, gelsolin and  **synapsin I**  has been attributed to their ability to disorganize the microtubule domains where most of the  **actin**  filaments are located.  Ref: 1570018 Nature, 1992
- **Synapsin I**  accelerates the initial rate of  **actin**  polymerization and increases the final steady state levels of polymerized  **actin** .  Ref: 8305899 J Physiol Paris, 1993
- Video microscopy experiments with purified components showed that rabies virus nucleocapsid inhibits the  **actin**  bundling effect induced by dephospho  **synapsin I** , a neuron specific protein which is known to exert a control on the  **actin**  based cytoskeleton.  Ref: 9367369 J Gen Virol, 1997

**Parallel relationship** (e.g. studied together, co-existance, homology, etc.)

- Under the conditions of our assay 0.45 microM  **synapsin I** , 4 microM F  **actin** , half maximal inhibition of  **actin**  binding and bundling by unphosphorylated  **synapsin I**  was found with 4.3 and 3.7 microM calmodulin, respectively.  Ref: 7849051 Biochemistry, 1995
- Thus, the state of association of  **synapsin I**  with synaptic vesicles, which is modulated by its state of phosphorylation, may affect  **actin**  assembly and the physico chemical characteristics of the synaptic vesicle microenvironment.  Ref: 8977109 FEBS Lett, 1996
- The  **actin**  binding activity of  **synapsin I**  phosphorylated by cAMP dependent protein kinase or by calmodulin dependent protein kinase II showed similar sensitivity to calmodulin inhibition to unphosphorylated  **synapsin I** .  Ref: 7849051 Biochemistry, 1995
- **Synapsin I**  is an  **actin**  bundling protein this activity is controlled by phosphorylation.  Ref: 7849051 Biochemistry, 1995
- Effects of the neuronal phosphoprotein  **synapsin I**  on  **actin**  polymerization.  Ref: 1597463 J Biol Chem, 1992
- The effect was observed at both low and high ionic strength, was specific for  **synapsin I** , and was still present when polymerization was triggered by F  **actin**  seeds.  Ref: 1317863 J Biol Chem, 1992
- This segment is adjacent to the  **actin**  binding domain and is within the region of the betaSpIISigmaI that we previously predicted as a candidate  **synapsin I**  binding domain based upon sequence homology.  Ref: 11033089 Brain Res, 2000
- We found that dephosphorylated  **synapsin I**  accelerates the initial rate of  **actin**  polymerization and decreases the rate of filament elongation.  Ref: 1317863 J Biol Chem, 1992
- **synapsin I**  is able to promote  **actin**  polymerization and bundling of  **actin**  filaments in the presence of synaptic vesicles.  Ref: 7876313 J Cell Biol, 1995
- Fluorescence approaches to the study of the  **actin**  nucleating and bundling activities of  **synapsin I** .  Ref: 8305899 J Physiol Paris, 1993
- By using fluorescence resonance energy transfer between purified components labeled with fluorescent probes, we now show that the binding of  **synapsin I**  to  **actin**  is a rapid phenomenon.  Ref: 8365471 FEBS Lett, 1993
- ii dephosphorylated synapsin II induces the formation of thick and ordered bundles of  **actin**  filaments with greater potency than  **synapsin I** .  Ref: 9517476 Eur J Neurosci, 1997
- We have analyzed the ability of  **synapsin I**  to interact with  **actin**  monomers and filaments using purified proteins derivatized with fluorescent probes.  Ref: 8305899 J Physiol Paris, 1993
- Dose response curves indicated that synapsin IIa was more potent than  **synapsin I**  in bundling  **actin**  filaments.  Ref: 7931311 J Neurochem, 1994
- MAP kinase dependent phosphorylation of  **synapsin I**  significantly reduced its ability to promote G  **actin**  polymerization and to bundle  **actin**  filaments.  Ref: 8622996 Proc Natl Acad Sci U S A, 1996
- **Synapsin I**  is a nerve terminal phosphoprotein which interacts with synaptic vesicles and  **actin**  in a phosphorylation dependent manner.  Ref: 8365471 FEBS Lett, 1993
- **Synapsin I**  bound either to synaptic vesicles or to pure phospholipid vesicles exhibits phosphorylation dependent  **actin**  nucleating activity.  Ref: 8977109 FEBS Lett, 1996
- By binding to both phospholipid and protein components of SV with high affinity and in a phosphorylation dependent fashion,  **synapsin I**  is believed to cluster SV and to attach them to the  **actin**  based cytoskeleton of the nerve terminal.  Ref: 9401959 J Physiol, 1997
- These observations suggest that  **synapsin I**  has a phosphorylation dependent nucleating effect on  **actin**  polymerization.  Ref: 1317863 J Biol Chem, 1992
- In the case of  **synapsin I** , these biological properties have been attributed to its ability to interact with both synaptic vesicles and the  **actin**  based cytoskeleton.  Ref: 9517476 Eur J Neurosci, 1997
- Here we show that calmodulin in the presence of calcium is a competitive inhibitor of both  **actin**  binding and bundling by  **synapsin I** .  Ref: 7849051 Biochemistry, 1995
- We have visualized the  **actin**  bundling activity of  **synapsin I**  using a non perturbing method, video enhanced microscopy of fluoresceinated  **synapsin I**  and  **actin**  filaments.  Ref: 8305899 J Physiol Paris, 1993
- Effects of the neuronal phosphoprotein  **synapsin I**  on  **actin**  polymerization.  Ref: 1317863 J Biol Chem, 1992
- the ability to cross link synaptic vesicles and  **actin**  is specific for  **synapsin I**  and is not shared by other basic proteins.  Ref: 7876313 J Cell Biol, 1995
- Synaptic vesicles depleted of endogenous  **synapsin I**  decrease the rate and the final extent of  **actin**  polymerization, an effect which is not observed with pure phospholipid vesicles.  Ref: 8977109 FEBS Lett, 1996
- Dephosphorylated  **synapsin I**  anchors synaptic vesicles to  **actin**  cytoskeleton an analysis by videomicroscopy.  Ref: 7876313 J Cell Biol, 1995
- Binding of  **synapsin I**  to  **actin**  can also be demonstrated when synaptic vesicles are present in the medium and appears to be modulated by ionic strength and  **synapsin I**  phosphorylation.  Ref: 8365471 FEBS Lett, 1993
- In the present paper we have analyzed the effects of  **synapsin I**  on the kinetics of  **actin**  polymerization and their modulation by site specific phosphorylation of  **synapsin I** .  Ref: 1317863 J Biol Chem, 1992
- In this study, we have examined whether or not calmodulin can regulate one of the activities of  **synapsin I** , namely, its interaction with F  **actin** .  Ref: 7849051 Biochemistry, 1995
- Rapid binding of  **synapsin I**  to F and G  **actin** .  Ref: 8365471 FEBS Lett, 1993
- We have found that synaptic vesicles have complex effects on the kinetics of  **actin**  polymerization, which vary depending on whether the synaptic vesicle specific phosphoprotein  **synapsin I**  is absent or present on their membrane.  Ref: 8977109 FEBS Lett, 1996
- The binding assay in the native state using antibodies specific to the tail region of  **synapsin I**  revealed that the binding is a high affinity with Kd of 9 nM, which is almost comparable to that of  **synapsin I**  to synaptic vesicles and to F  **actin** .  Ref: 9070909 Biochem Biophys Res Commun, 1997
- Brain beta spectrin contains three structural domains and we suggest the position of several functional domains including f  **actin** ,  **synapsin I** , ankyrin and spectrin self association sites.  Ref: 8479293 Brain Res Mol Brain Res, 1993
- Dephosphorylated  **synapsin I**  was also able to induce  **actin**  polymerization and bundle formation in the absence of KCl and MgCl2.  Ref: 1317863 J Biol Chem, 1992
- **Synapsin I**  appears to bind G  **actin**  with a very high stoichiometry 1 2 4, and the complex behaves as an F  **actin**  nucleus, producing  **actin**  filaments under conditions where spontaneous polymerization is negligible.  Ref: 1597463 J Biol Chem, 1992
